# Supplementary material for: WDR82-Mediated H3K4me3 Is Associated with Tumor Proliferation and Therapeutic Efficacy in Pediatric High-Grade Gliomas
Source: Cancers (Basel). 2023 Jun 30;15(13):3429. doi: 10.3390/cancers15133429 (PMC10340597; doi:10.3390/cancers15133429)
Supplement: Supplementary file 1 [file cancers-15-03429-s001.zip › cancers-2365551-supplementary.pdf]

Supplementary Materials

WDR82 mediated H3K4me3 is associated with tumor proliferation and therapeutic efficacy in pediatric high-grade gliomas

Nitin Wadhwani,[1] Sonali Nayak,[2] Yufen Wang,[3] Rintaro Hashizume,[4*] Chunfa Jie,[5] Barbara Mania-Farnell,[6] Charles David James,[7] Guifa Xi,[2,7] Tadanori Tomita,[2,7]

1. Department of Pathology, Ann & Robert H. Lurie Children's Hospital of Chicago, Northwestern University Feinberg School of Medicine, Chicago, IL 60611 USA
2. Division of Pediatric Neurosurgery, Ann & Robert H. Lurie Children's Hospital of Chicago, Northwestern University Feinberg School of Medicine, Chicago, IL 60611 USA
3. Department of Radio-oncology, Northwestern University Feinberg School of Medicine, Chicago, IL 60611 USA
4. Department of Pediatrics, Northwestern University Feinberg School of Medicine, Chicago, IL 60611 USA
5. Department of Biochemistry and Nutrition, Des Moines University Medicine and Health Sciences, Des Moines, IA 50312 USA
6. Department of Biological Sciences, Purdue University Northwest, Hammond, IN 46323 USA
7. Department of Neurological Surgery, Northwestern University Feinberg School of Medicine, Chicago, IL 60611 USA

***Current institution:** Division of Pediatric Hematology/Oncology, Department of Pediatrics, University of Alabama at Birmingham, 1600 7th Avenue South, Birmingham, AL 35233 USA

Corresponding author
Guifa Xi, MD, PhD
The Department of Neurological Surgery, Northwestern University Feinberg School of Medicine
Address: Simpson Querrey Biomedical Center, Room 6-500
303 Superior Street, Chicago, IL 60611
Phone: (312) 503 4296
Email: xiguifa@yahoo.com

Tadanori Tomita, MD
Division of Pediatric Neurosurgery, Ann & Robert H. Lurie Children's Hospital of Chicago, Northwestern University Feinberg School of Medicine
Address: 225 E Chicago Ave, Box 28
Chicago, IL 60611
Phone: (312) 227 4220
Email: ttomita@luriechildrens.org

Supplementary Materials and Methods

Tumor sphere formation assay. Cells were cultured in T25 flasks with or without Dox (2μg/ml), incubated at 37°C with 5% CO2 for three days, harvested as single cell suspension and counted prior to plating 2000 cells/well in ultra-low attachment 24-well plates (Cat#3473, Corning Incorporated). Cell culture medium contained DMEM:F12 (1:1) (Cat#12660-012), B27 (Cat#A35828-01), EGF (Cat#PHG0311) 20ng/ml, b-FGF (Cat#PHG0021) 20ng/ml from Gibico. Cell growth was monitored daily, images were captured and the number of spheres were counted with EVOS FL microscopy (Life Technologies) at day 3.

MTS assay for chemosensitivity assessment. pHGG cells were used to determine if reducing WDR82 through inducible knockdown affected cell viability in the absence or presence of chemotherapeutic drugs, vincristine (VCR) and cisplatin (CDDP). For the cells in the absence of chemotherapeutic drugs, 1×104 cells/100μl were plated in 96-well plates with complete cell culture medium with or without Dox (2μg/ml), and subjected to 3-(4, 5-dimethylthiazol-2-yl)-5-(3-carboxymethoxyphenyl)-2-(4-sulfophenyl)-2H-tetrazolium (MTS, Promega) assay. Cells in the presence of chemotherapeutic drugs, were harvested and counted with TC-20TM Cell Counter (Bio-Rad Laboratories Inc.), equal numbers of cells were plated in 96-well plates with complete cell culture medium in the absence or presence of (Dox) (2μg/ml) for 2 days following treatment with cisplatin (CDDP, 10-3 to 103 μM), and subjected to MTS assay after 72h.

Radiation therapy. Cells were seeded into 6-well tissue culture plates and allowed to adhere. Attached cells were irradiated (0, 1, 2 or 4Gy) and treated with or without Dox (SKU#D9891-10G, Sigma-Aldrich) at 2μg/ml 2 hours after irradiation. Radiation was delivered by a 137Cs source (Mark I, model 68A irradiator, JL Shepherd & Associates) as previously described.1 Cells were incubated with Dox for 2 weeks, at which time colonies were stained with methylene blue (0.66% solution in 95% ethanol) and counted. Plating efficiencies were calculated as the ratio of the number of colonies formed to the number of cells seeded. Colonies of >50 cells were counted for surviving fraction determinations. Surviving fractions were calculated as the plating efficiency of treated cells divided by the plating efficiency of control cells, as previously described.2 Dose enhancement factors were calculated at 10% survival.

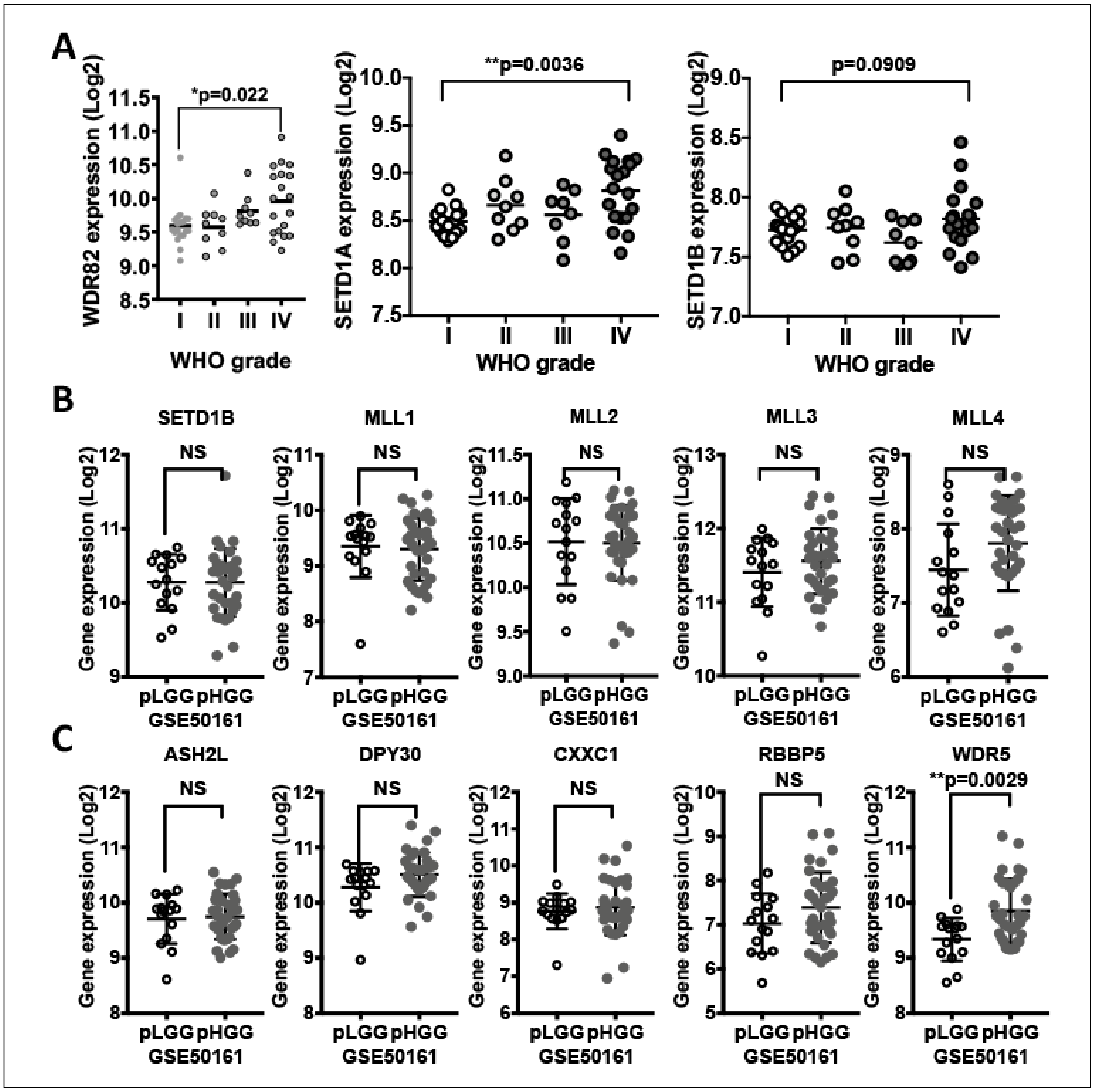

Supplementary Figure S1. In silico analysis of expression of subunits of human SET-COMPASS complexes in pediatric gliomas. A. Expressions of WDR82, SETD1A and SETD1B in pediatric gliomas. B and C. In silico analysis of GEO dataset GSE50161 showing expression of SETD1B and MLL1-4 (B) and expression of SETD1A/B subunits in pediatric low- (pLGG) and high- (pHGG) grade gliomas (C).

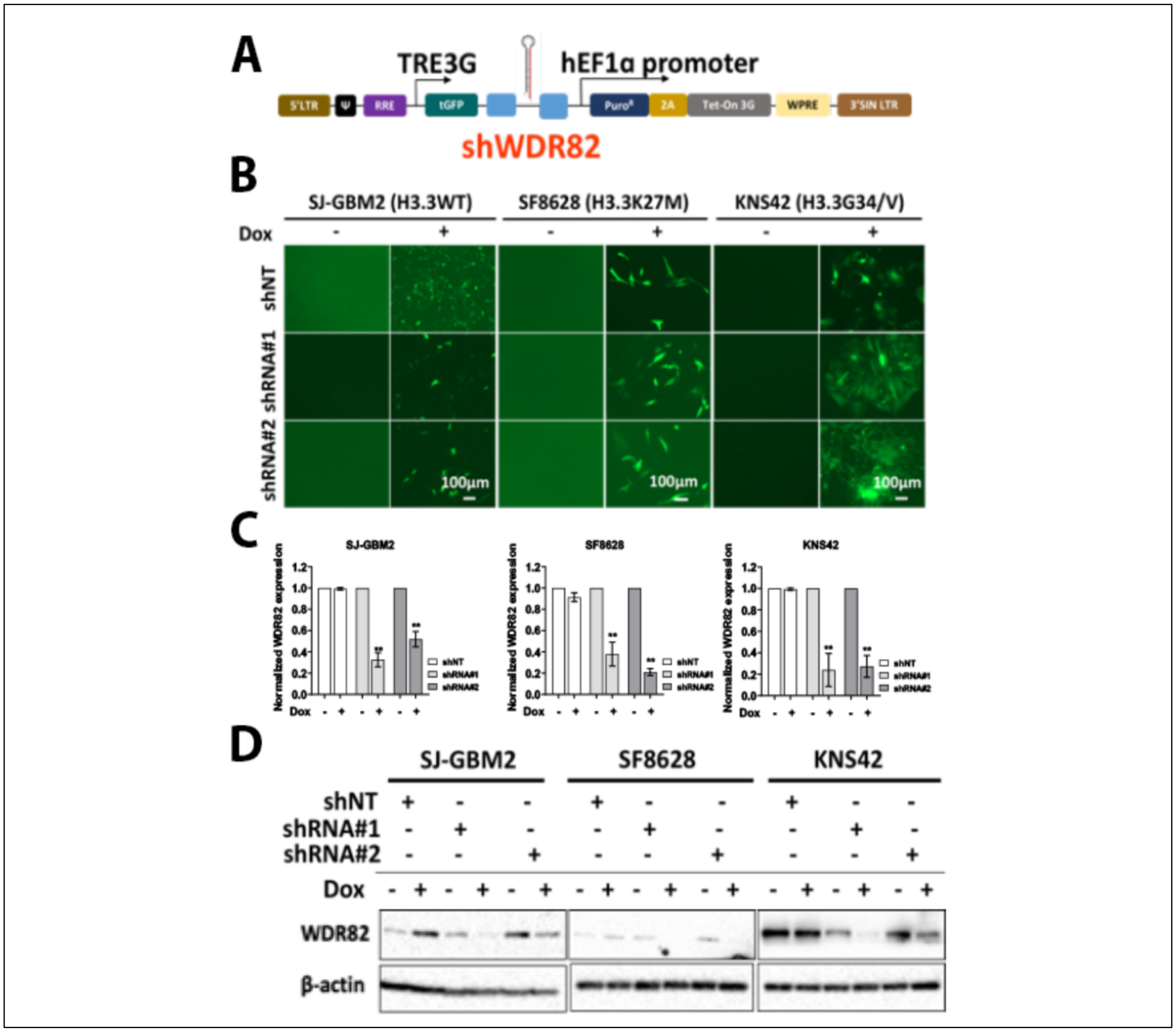

Supplementary Figure S2. Establishment and validation of a WDR82 expression inducible knockdown system. A. The illustration indicates the main components of DOX inducible small hairpin WDR82 (shWDR82) lentiviral vectors. B. Representative images show SJ-GBM2, SF8628 and KNS42 cells transduced with non-target (shNT, negative control) or small hairpin RNAs (shRNA#1 and shRNA#2) against WDR82 in the absence or presence of 2μg/ml Dox at day 5. Real-time PCR (C) and western blot (D) results using RNA and protein from cells in B.

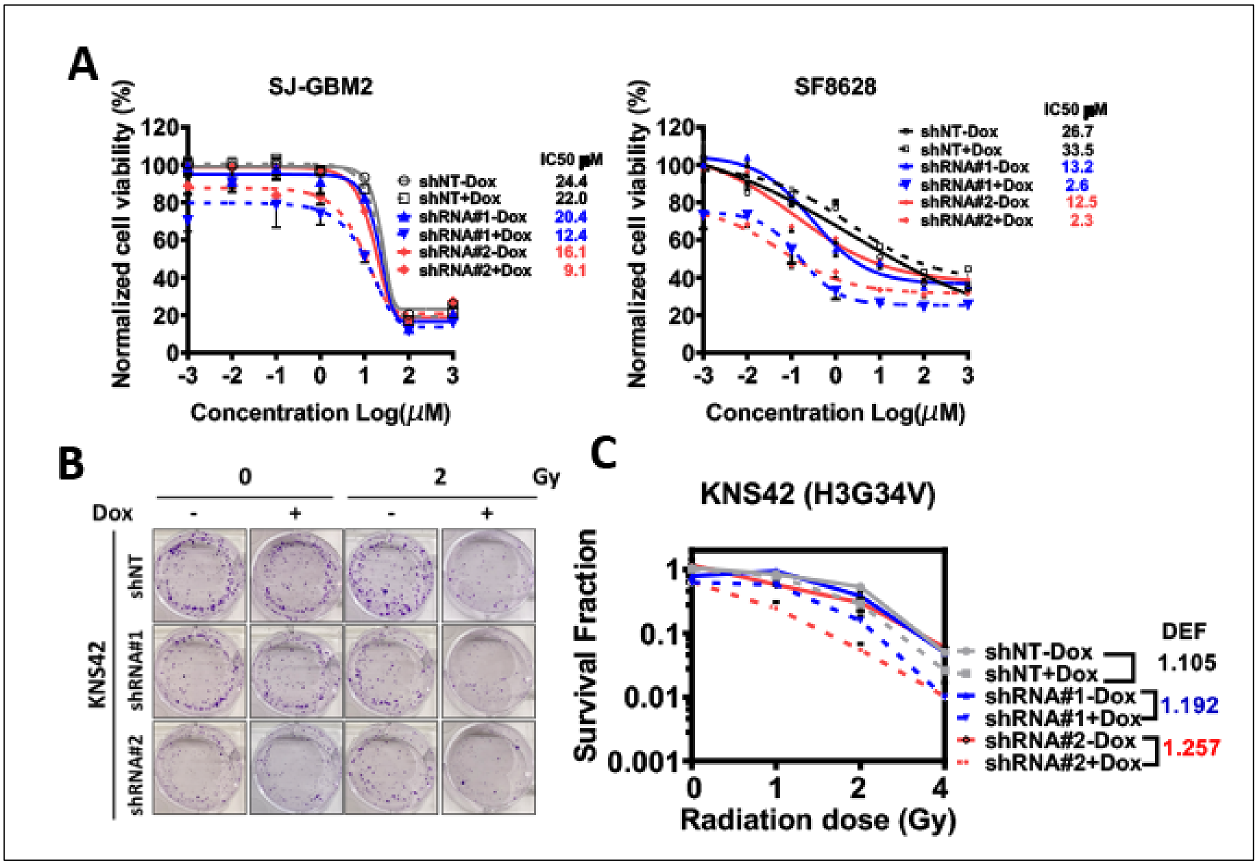

Supplementary Figure S3. WDR82 knockdown increases chemotherapeutic and radiation sensitivity. A. Normalized cell viability of SJ-GBM2 and SF8628 cells transduced with inducible shRNAs #1 and #2 against WDR82 and control non-target shRNA (shNT) in the absence or presence of Dox, 2μg/ml, following treatment with cisplatin (CDDP). B. Representative images show colonies from 1000 cells in the absence (-) or presence (+) of Dox, following radiation therapy. (C) Quantitative results from B.

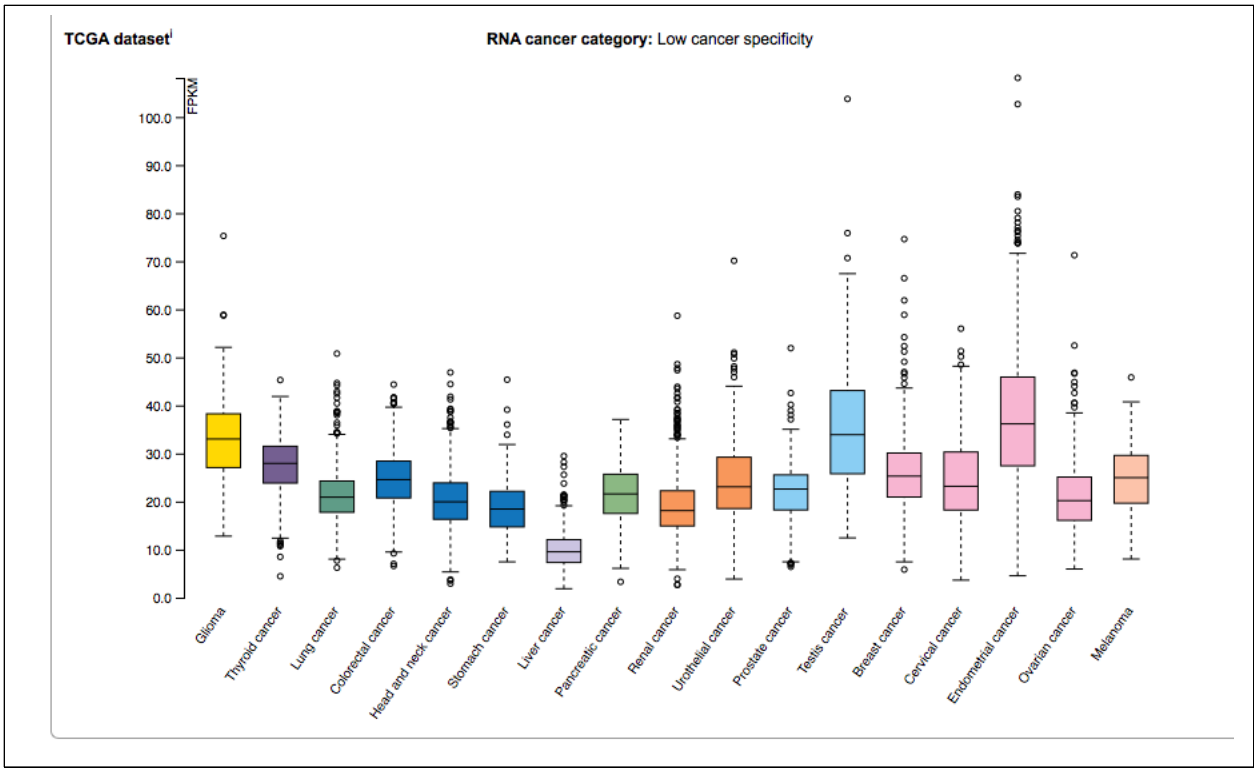

Supplementary Figure S4. WDR82 expression in human cancers presented with TCGA dataset (https://www.proteinatlas.org/ENSG00000164091-WDR82/pathology).

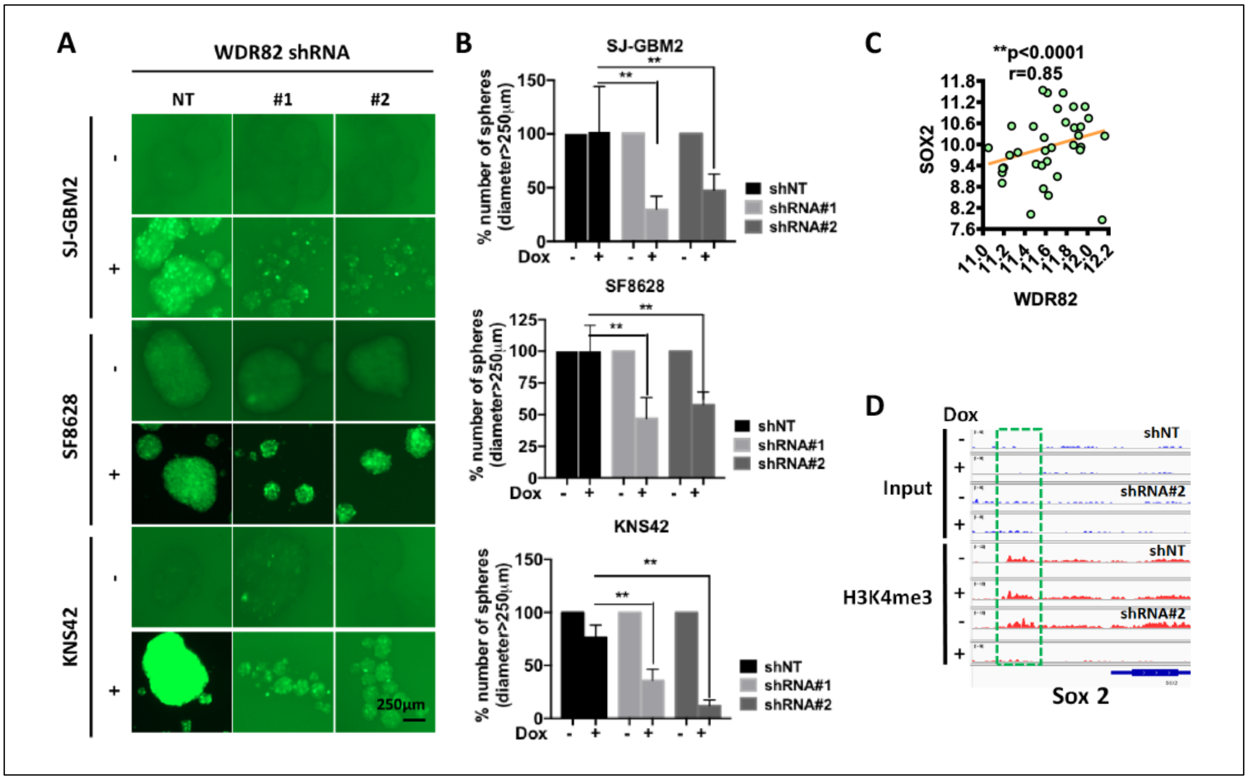

Supplementary Figure S5. WDR82 is associated with stem cell characteristics. Representative images (A) and normalized quantitative results for sphere diameter > 250µm (B), show tumor sphere formation in SJ-GBM2 (H3WT), SF8628 (H3K27M) and KNS42 (H3G34V) pHGGs transduced with control shNT, or shRNA#1, or shRNA#2 against WDR82, in the absence (-) and presence (+) of Dox (2µg/ml). C. Correlation between WDR82 and SOX2 through in silico analysis of dataset GSE50161. (D) ChIP seq results from SJ-GBM2 cells show H3K4me3 level at the SOX2 promoter. SJ-GBM cells non-treated or treated with Dox, 2µg/ml.

Supplementary Table S1 Clinicopathological information for pediatric primary gliomas IHC stained for H3K4me3.

Variable	Category	N (%)	H3K4me3					*P* value
			0	1	2	3	4	
Gender	female	27 (48.2)	12 (48.0)	4 (44.4)	8 (57.1)	2 (33.3)	1 (50.0)	0.9047
	male	29 (51.8)	13 (52.0)	5 (55.6)	6 (42.9)	4 (66.6)	1 (50.0)	
Age	<=3 y	8 (14.3)	2 (8.0)	5 (50.0)	1 (7.1)	0 (0.0)	0 (0.0)	0.0114
	>3y	48 (85.7)	23 (92.0)	5 (50.0)	13 (92.9)	5 (62.5)	2 (100.0)	
Tumor location	Supratentorial	11 (19.6)	1 (4.0)	3 (30.0)	6 (42.9)	1 (12.5)	0 (0.0)	0.0924
	Infratentorial	23 (41.1)	10 (40.0)	4 (40.0)	5 (35.7)	2 (25.0)	2 (100.0)	
	Midline (including spinal cord)	22 (39.3)	14 (56.0)	3 (30.0)	3 (21.4)	2 (25.0)	0 (0.0)	
WHO grade	Low (grade I & II)	46 (82.1)	26 (100.0)	8 (100.0)	10 (71.4)	2 (25.0)	0 (0.0)	<0.0001
	High (grade III & IV)	10 (17.9)	0 (0.0)	0 (0.0)	4 (28.6)	4 (50.0)	2 (100.0)	

P value was calculated by Fisher's Exact test.
